# Clinical Bacteriology and Hæmatology for Practitioners

**Published:** 1906-09

**Authors:** 


**Clinical Bacteriology and Hæmatology for Practitioners.** By W. D'Este Emery, M.D. Pp. xiii., 240. London: H. K. Lewis. 1906.

This book, which is a second edition of "A handbook of bacteriological diagnosis for practitioners," is exceedingly useful so far as it goes. In the third edition, perhaps, the author will extend it so as to include the remaining clinical methods, which the practitioner may have occasion to use. Some time, perhaps, the present-day faith in the inviolable judgment, &c., of the so-called "research associations"—such is the dignity of research —may waver, and the medico may turn his own knowledge to effect. Anyone whose mind is already thus inclined, may be recommended to purchase this book. The directions for work are given in concise and precise terms. Whoso fulfils these will attain good results. In addition, there are some valuable notes upon the interpretation of the figures and appearances obtained.

It would be invidious to cavil at the omission of many subjects. When the reader has mastered the details of the necessary apparatus and processes of the laboratory diagnosis of diphtheria, tetanus, pneumonia, influenza, anthrax, tubercle, leprosy, actinomycosis, glanders, typhoid, gonorrhœa, syphilis, cholera, plague, soft sore, ringworm, skin diseases, the various secretions, and exudates and the blood, it will be time to take up those methods, which must often be turned to for confirmation of the simpler and preliminary tests herein contained.

The illustrations are instructive and well designed. There are, however, as in similar books, a number of cuts from various catalogues, for whose inclusion the several instrument makers, &c., ought to be duly thankful. How long shall the author advertise the "apparatus dealer" without adequate remuneration? The practitioner must not be unequally yoked with an unqualified assistant. Why should our makers of books disfigure their pages with illustrations from the catalogues of the Phœnicians?

